# Exploring the Interplay Between Self‐Identity, Affective Style, Emotion Regulation, and Anxiety: Based on Bayesian Network Model

**DOI:** 10.1002/brb3.70290

**Published:** 2025-02-09

**Authors:** Ruizhi Huang, Huiqing Shen, Ye Yuan, Ke Jiang, Zhilin Wang

**Affiliations:** ^1^ Department of Surgery West China Hospital of Sichuan University Chengdu Sichuan China; ^2^ School of Mental Health Wenzhou Medical University Wenzhou China; ^3^ Department of Mathematics and Statistics Chonnam National University Gwangju Republic Korea; ^4^ Teacher Education College Lishui University Lishui Zhejiang Province China; ^5^ Center for Brain, Mind and Education Shaoxing University Shaoxing China; ^6^ Mental Health Education and Research Center, School of Marxism Nanjing Medical University Nanjing China

**Keywords:** affective style, anxiety, Bayesian network model, emotion regulation, intolerance of uncertainty (IU), network analysis, self‐identity

## Abstract

**Objective:**

This study aimed to explore the complex relationships between self‐identity, affective style, emotion regulation, and intolerance of uncertainty (IU) in predicting anxiety. A model was proposed to integrate these factors, investigating their combined influence on anxiety.

**Method:**

Involving 608 university students who completed self‐report measures of self‐identity, affective style, emotion regulation, IU, and anxiety. Network analysis and Bayesian network modeling were used to identify direct and mediating effects among these variables.

**Results:**

Network analysis revealed that self‐identity, affective style, and IU directly predicted trait anxiety, with adjusting affective style emerging as a central factor. Bayesian network modeling further showed that IU and affective style mediated the impact of self‐identity on anxiety. Notably, emotion regulation did not mediate the relationship between affective style and anxiety, suggesting a possible spurious correlation. The model achieved a predictive accuracy of 90.13% for trait anxiety and 88.49% for state anxiety.

**Conclusion:**

The findings highlight the central role of self‐identity in anxiety interventions, while also emphasizing the importance of addressing affective styles and IU. The results suggest that emotion regulation strategies alone may not directly reduce anxiety, indicating a need for more comprehensive clinical approaches.

## Graphical abstract

This study employed network analysis and Bayesian network modeling to investigate the complex interplay among self‐identity, affective style, emotion regulation, intolerance of uncertainty (IU), and anxiety in 608 university students. Findings reveal that self‐identity, affective style, and IU directly predict anxiety, with affective style playing a central role. IU and affective style mediate self‐identity's impact on anxiety, while emotion regulation's effect appears spurious. The model achieved high predictive accuracy for both trait and state anxiety, highlighting the importance of targeting self‐identity, affective styles, and IU in anxiety interventions.

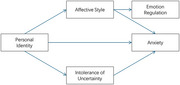

## 1 Introduction

### 1.1 Background Information

Anxiety is a complex psychological response to perceived threats or uncertainties, often characterized by fear, tension, and unease (American Psychiatric Association 2013). Traditionally, anxiety has been studied through cognitive–behavioral and neurobiological lenses, focusing on physiological arousal, cognitive distortions, and behavioral responses. More recently, theorists have emphasized the dynamic and context‐sensitive nature of psychological experiences, highlighting how individuals’ interactions with their environment shape emotional responses (Barrett 2017). They propose that cognition and emotion are fluidly constructed through the dynamic interaction between the individual and the external world (Barrett 2017; Scarantino and de Sousa 2021; Shapiro and Spaulding 2014). Some researchers, such as Shapiro and Spaulding, posit that emotions, including anxiety, emerge through continuous exchanges between the individuals and their surroundings, suggesting that psychological experiences are fluid and adaptive rather than static (Shapiro and Spaulding 2024).

This study would like to explore how anxiety is influenced by self‐identity, affective style, and emotion regulation. These psychological factors are closely tied to an individual's ability to tolerate uncertainty, which is a key precursor to anxiety (Grupe and Nitschke 2013). By utilizing advanced methods such as network analysis and Bayesian network models, this study aims to uncover the complex relationships between these factors and provide insights into anxiety regulation.

### 1.2 Interaction Perspective on Anxiety

From an interaction perspective, anxiety is not simply a physiological or cognitive reaction but a psychological process that is co‐constructed through the interplay between an individual and the external world. According to this theory, individuals interpret and respond to external stimuli based on their internal constructs, which are shaped by self‐identity, emotion regulation, and their tolerance of uncertainty (Lindquist et al. 2022).

Erik Erikson conceptualized self‐identity in psychology, defining it through four interactive aspects: an individual's conscious awareness, the pursuit of continuity in personal characteristics, a criterion for self‐integration, and an inner resonance with the group's ideals and identity (Erikson 1956). Continuing in the psychoanalytic tradition, Erikson argued that the self is closely related to anxiety, which arises from a crisis of self‐identity. This crisis occurs when an individual fails to effectively integrate and coordinate diverse experiences into a cohesive self (Erikson 1956). A stable and well‐defined self‐identity provides a framework that helps individuals reduce uncertainty and mitigate anxiety, a view supported by Kelly (1955), who emphasized that coherence in self‐identity is crucial for alleviating psychological discomfort and preventing anxiety (Kelly 1955). Conversely, individuals with a fragmented or weak sense of identity are more susceptible to uncertainty. They struggle to manage new experiences and predict outcomes, which heightens their anxiety (Abraham, Utami, and Faza 2014; Hayward et al. 2020). In summary, self‐identity plays a central role in the interaction model of anxiety (Lillevoll, Kroger, and Martinussen 2013).

Intolerance of uncertainty (IU) is another key factor in understanding anxiety from this framework. IU refers to an individual's difficulty in accepting the ambiguity of future outcomes, which can lead to increased anxiety, especially in situations involving uncertainty (Grupe and Nitschke 2013). Individuals with high IU find it difficult to integrate new or unpredictable information into their cognitive models, which disrupts the individuals and their surroundings interaction process and exacerbates anxiety (Boswell et al. 2013).

Affective style, or habitual patterns of emotional response, also plays a critical role in how individuals experience and regulate anxiety. Affective styles can be categorized into three distinct types, each with unique characteristics and applications in real‐world scenarios. The first style, known as reappraisal, involves readjusting emotions to effectively adapt to situational demands by altering one's emotional reaction through a change in perspective about the situation. The second strategy aims to conceal or suppress emotions, inhibiting the outward expression of emotions without altering the internal emotional experience. Although suppression can sometimes be socially appropriate and necessary, it often incurs a psychological cost, including increased stress and decreased well‐being, due to the ongoing experience of suppressed negative emotions. The third approach involves tolerating and accepting all emotions, including those that are unwanted or aversive. Characterized by an open and nonjudgmental stance toward emotional experiences, acceptance promotes better psychological resilience (Hofmann and Kashdan 2010). Adaptive affective styles, characterized by emotional flexibility and positive regulation strategies, enhance an individual's ability to tolerate uncertainty and effectively manage anxiety (Davidson 1992). Conversely, rigid or maladaptive affective styles may limit an individual's capacity to cope with uncertainty, thereby increasing anxiety (Gross and Thompson 2014; Gross 2013b). The interaction between affective style and emotion regulation is essential in determining how individuals respond to anxiety‐inducing situations, particularly in the face of uncertainty.

Emotion regulation strategies are crucial for managing anxiety within this framework. Researchers have found a significant correlation between emotion regulation and anxiety. For example, adaptive strategies, such as cognitive reappraisal, allow individuals to reinterpret uncertainty in a manageable way, thereby reducing anxiety (Gross 2013a, 2013b). Conversely, maladaptive strategies, like emotional suppression, can increase anxiety by preventing the integration of new information and maintaining a focus on perceived threats (Lenzo et al. 2021).

### 1.3 Research Gaps and Hypotheses

Although existing research has separately explored the relationships between self‐identity, IU, affective style, and emotion regulation with anxiety, a dynamic model based on the interaction between the individual and the environment to explain the development of anxiety is still lacking. Additionally, there remains a shortage of empirical research that comprehensively examines how these factors interact. The mediating role of intolerance of uncertainty in these relationships has not been fully explored.

Based on a review of previous research, we believe that the constructs of self‐identity, affective style, emotion regulation, and intolerance of uncertainty are not only individually important for understanding anxiety but also form a dynamic system that co‐constructs the emotional experience of anxiety. We propose a hypothetical model that integrates the interaction of these factors. Recent research suggests that anxiety development involves complex interactions between personality traits and regulatory processes (Yang et al. 2023; Zhao et al. 2024). Empirical evidence has demonstrated that individuals with a stronger sense of self‐identity show greater resilience in uncertain situations and are more likely to employ adaptive emotion regulation strategies (Breakwell 2021, 2023).

Anxiety often arises when there is a mismatch between an individual's internal expectations and external realities, particularly in uncertain situations. This discrepancy triggers cognitive processes aimed at reducing uncertainty, and if these processes are unsuccessful, anxiety is likely to intensify. Self‐identity serves as a key reference point in perceiving environmental uncertainty during the individual–environment interaction (Hogg 2007, 2021), making it a primary factor in the development of anxiety. Previous studies have shown that individuals with a clear sense of self‐identity demonstrate lower levels of anxiety when faced with uncertainty (Hirsh and Kang 2016), suggesting that self‐identity may serve as a psychological anchor that helps individuals navigate ambiguous situations.

Compared to self‐identity, IU, affective style, and emotion regulation are more context dependent, thus acting as mediators between self‐identity and anxiety. Between affective style and emotion regulation, affective style exhibits a more dispositional tendency (Davidson 1992), while emotion regulation is more expressive (Gross 1998). Therefore, affective style and emotion regulation form a chain mediation relationship between self‐identity and anxiety. IU is a dispositional trait that manifests in the interaction between the individual and the environment, thus serving a mediating function between self‐identity and anxiety. The research hypotheses are as follows:

H1: Self‐identity predicts levels of anxiety, with a stronger sense of identity associated with lower anxiety.
H2: Intolerance of uncertainty mediates the relationship between self‐identity and trait anxiety, with higher IU leading to increased anxiety in individuals with weaker personal identities.
H3: Adaptive emotion regulation strategies, such as cognitive reappraisal, predict lower levels of anxiety, while maladaptive strategies, such as emotional suppression, are associated with higher anxiety. Affective style forms a mediation relationship between self‐identity and anxiety.
H4: Emotion regulation mediates the relationship between affective style and anxiety, with more adaptive affective styles linked to lower anxiety through the use of effective emotion regulation strategies. Emotion regulation forms a mediation relationship between affective style and anxiety.

### 1.4 Application of Network Analysis and Bayesian Network Model

This study seeks to apply network analysis and the Bayesian network model to explore the complex relationships between self‐identity, affective style, emotion regulation, and anxiety.

Network analysis is particularly suitable for mapping direct and indirect relationships between constructs, providing a visual representation of the centrality and interaction of variables like self‐identity and IU (Epskamp, Borsboom, and Fried 2018). This method can highlight how the weakening of self‐identity coherence or the maladaptive use of emotion regulation strategies might lead to higher levels of anxiety by disrupting the ability to tolerate uncertainty.

The Bayesian network model, on the other hand, offers several advantages over traditional linear models. Unlike regression‐based models, which assume fixed relationships between variables, Bayesian network models incorporate uncertainty and allow for the probabilistic exploration of cause‐and‐effect relationships (Pearl 2009). This method is well suited for examining complex psychological constructs, as it can handle the nonlinear interactions and dependencies that are inherent in processes like anxiety formation (McNally 2016).

By employing these methodologies, this study aims to provide a deeper understanding of the mechanisms underlying anxiety and offer potential avenues for personalized and effective interventions in anxiety management.

## 2 Methods

### 2.1 Study Design

Because the research aims to explore the factors influencing anxiety in the general population, independent of individual life cycle changes, this study employed a cross‐sectional research design to investigate the relationships between self‐identity, affective style, emotion regulation, intolerance of uncertainty (IU), and anxiety. Network analysis and Bayesian modeling were used to examine the direct and mediating effects of these variables on anxiety.

In this study, we primarily employ two data analysis methods. First, we utilize an adaptive LASSO network that encompasses five scales with a total of nine nodes. Considering the potential connections, the number is calculated as 9 × (9 − 1)/2 = 36. Therefore, the estimated parameter count includes 36 connections plus 9 node thresholds, totaling 45 parameters. It is generally recommended that the sample size be two to three times the number of parameters, suggesting a minimum required sample size of 45 × 3 = 135 for our analysis. On the other hand, Bayesian methods are less demanding in terms of sample size. Typically, a simple model requires at least 100 samples, while more complex models need over 300 samples. Based on these guidelines, our study is designed to collect no fewer than 300 data samples during the planning stage.

### 2.2 Participants

A total of 681 participants were recruited using convenience sampling from universities in the Chinese cities of Wenzhou and Nanjing. The sample included undergraduate, master's, and doctoral students. After excluding 74 questionnaires due to incomplete responses or incorrect answers to validity check questions (e.g., selecting “Yes” for “I was born before 1920”), the final sample comprised 607 valid questionnaires. The demographic characteristics of the participants are shown in Table 1.

**TABLE 1:** Demographic information table.

Condition	Category	Frequency	Ratio (%)
**Age**	≤20	264	43.49
	21–25	286	47.12
	26–30	48	7.91
	31–35	4	0.66
	36–40	5	0.82
**Gender**	Male	247	33.77
	Female	360	66.23
**Education**	Undergraduate	528	86.99
	Master	50	8.24
	Doctor	29	4.78

There are two reasons for selecting university students as participants: first, university students possess strong cognitive abilities, allowing them to accurately understand the content of the questionnaires and respond appropriately; second, while they have relatively mature minds, they are less influenced by professional training or other life events, making their psychological mechanisms representative of the general characteristics of normal adults. Therefore, when aiming to study the general psychological mechanisms of “human,” recruiting university students as participants has its advantages.

### 2.3 Procedures

Data were collected via online surveys during the final exam period in December 2022. Well‐trained students administered standardized questionnaires to participants at designated university locations. Prior to completing the questionnaires, participants were informed about the confidentiality of their responses and the importance of honest answers. The survey took approximately 10 min to complete. Both participants and their instructors provided signed informed consent. The collected data were examined for errors, and questionnaires showing obvious response patterns (e.g., nonsensical answers) were removed.

To achieve the research objectives, we sought participants who were experiencing anxiety levels higher than their usual average. In some experimental studies, researchers often design specific tasks to induce anxiety in participants. We chose to conduct the survey during exam week, utilizing the fact that students typically experience elevated levels of anxiety during this period to meet the requirements of the study.

### 2.4 Measures

#### 2.4.1 State‐Trait Anxiety Inventory

The State‐Trait Anxiety Inventory (STAI) is a widely recognized 40‐item self‐report questionnaire developed to measure both state anxiety (S‐AI; 20 items) and trait anxiety (T‐AI; 20 items) (Spielberger et al. 1983 ). Participants respond to items using a 4‐point Likert scale ranging from 1 (“not at all”) to 4 (“very much so”), with statements such as “我感到心情平静 (I feel calm)” and “我的思想处于混乱状态 (I have disturbing thoughts).” Historically, the scale has demonstrated robust reliability, with Cronbach's *α* ranging from 0.82 to 0.95 for state anxiety and 0.91 for trait anxiety (Spielberger et al. 1971). The Chinese version further supports these findings, showing correlation coefficients of 0.88 for S‐AI and 0.90 for T‐AI. The correlation between S‐AI and T‐AI was 0.84 in the initial assessment and 0.77 in a subsequent test, confirming satisfactory consistency (Zheng et al. 1993). In the current study, the entire scale exhibited exceptional internal consistency with a Cronbach's *α* of 0.959, while the subscales reported 0.932 for S‐AI and 0.911 for T‐AI (calculated by JASP, same as the following).

#### 2.4.2 Emotion Regulation Questionnaire

The Emotion Regulation Questionnaire (ERQ) evaluates two core dimensions of emotion regulation: cognitive reappraisal and expressive suppression. It comprises 10 items rated on a 7‐point Likert scale (1 = “strongly disagree” to 7 = “strongly agree”) (Gross 2013b ). Sample items include statements such as, “当我想多感受到积极的情绪的时候, 比如高兴或兴趣, 我改变我所考虑的事情 (When I want to feel more positive emotion (such as joy or amusement), I change what I'm thinking about).” The original reliability of the ERQ has been reported as 0.75 to 0.82 for cognitive reappraisal and 0.68 to 0.76 for expressive suppression (Gross and John 2003). In the Chinese version, Wang et al. reported a test–retest reliability of 0.82 and an internal consistency reliability of 0.85 for cognitive reappraisal (Wang et al. 2007). For expressive suppression, the test–retest reliability and internal consistency reliability were reported as 0.79 and 0.77, respectively. In the current study, the Cronbach's *α* for the overall ERQ was 0.814, with subscale *α* values of 0.833 for cognitive reappraisal and 0.727 for expressive suppression.

#### 2.4.3 Affective Style Questionnaire

The Affective Style Questionnaire (ASQ) (Kurilshikov et al. 2021) consists of 20 items in the Chinese version and 19 items in the original English version (Hofmann and Kashdan 2010; Liu, Zhou, and Zhou 2011), measuring three dimensions of affective style: concealing, adjusting, and tolerating (Davidson 1992; Hofmann and Kashdan 2010 ). Participants rate their agreement with each item on a 5‐point Likert scale, ranging from 1 (“strongly disagree”) to 5 (“strongly agree”). An example item from the Chinese version is: “别人通常看不出来我心里是怎么想的 (People usually can't tell how I am feeling inside).” However, the items in the Chinese and English versions are not directly equivalent. The Chinese version found a Cronbach's *α* of 0.73 for the overall scale, with subscale values of 0.79 for concealing, 0.76 for adjusting, and 0.60 for tolerating. Test–retest reliability coefficients for the Chinese version were 0.77, 0.74, and 0.84 for concealing, adjusting, and tolerating, respectively (Liu, Zhou, and Zhou 2011). In this study, the overall Cronbach's *α* was 0.889, with subscale values of 0.856 for concealing, 0.815 for adjusting, and 0.509 for tolerating.

#### 2.4.4 Self‐Identity Scale

The Self‐Identity Scale (SIS), developed by Ochse and Plug in 1986, assesses the strength and coherence of an individual's self‐identity, aligning with Erikson's theoretical framework on identity development. This scale comprises 19 items, including statements such as “我不清楚自己是怎样的人 (I am not sure what kind of person I am)” and “别人对我的看法总是改变 (Others’ opinions of me always change).” Responses are collected using a 4‐point Likert scale, ranging from 1 (“completely disagree”) to 4 (“completely agree”), where higher scores denote a stronger and more coherent self‐identity. The reliability of the Chinese version was reported with a Cronbach's  *α* of 0.727 (Li and Lou 2009). In this study, the Cronbach's *α* for the SIS was 0.786.

#### 2.4.5 Intolerance of Uncertainty Scale

The Intolerance of Uncertainty Scale (IUS‐12) is a 12‐item scale that measures an individual's capacity to tolerate uncertainty, employing a 5‐point Likert scale ranging from 1 (“completely disagree”) to 5 (“completely agree”) (Carleton, Norton, and Asmundson 2007). The scale includes items such as “无法预料的事情会让我心烦意乱 (Unforeseen events upset me greatly).” Higher scores on the IUS‐12 signify greater intolerance of uncertainty. The scale has shown excellent internal consistency, with Cronbach's *α* values typically exceeding 0.90 (Huntley et al. 2020). For the Chinese version of the scale, the total score reliability ranged from 0.704 to 0.878, and the test–retest reliability was between 0.695 and 0.78 (Zhang et al. 2017). In the current study, the Cronbach's  *α* was 0.829, indicating strong internal consistency.

### 2.5 Data Analysis

Data and network analyses for this study were conducted using JASP 0.16.2.0, R, and R Studio. Based on the data analysis results, a Bayesian network model was developed using GeNIe 3.0 Academic.

#### 2.5.1 Descriptive Statistics

Mean scores, standard deviations, and correlation matrices were calculated for all variables to provide a basic overview of the relationships between self‐identity, affective style, emotion regulation, IU, and anxiety.

#### 2.5.2 Network Analysis

##### 2.5.2.1 LASSO Network

The R programming language and R Studio were used to construct an adaptive Least Absolute Shrinkage and Selection Operator (LASSO) network analysis; packages such as Bootnet, Networktools, and Qgraph were used for an in‐depth analysis of the relative strengths of factors potentially associated with anxiety. The network's design represents varying weights through line thickness, with thicker lines indicating stronger correlations.

##### 2.5.2.2 Centrality Indices

The centrality indices were calculated using the Centrality Plot function in R and R Studio's graph packages. These indices help identify the relative importance of nodes within the network structure. As noted by Bringmann et al. (2019), nodes with high centrality tend to exert a strong influence on other factors within the network.

##### 2.5.2.3 Robustness

To ensure the robustness of the network, its validity was assessed using the Bootnet package, with a focus on correlation stability coefficients (CS coefficients). Ideally, a CS coefficient should exceed 0.25, with values above 0.50 preferred for reliable stability (Epskamp, Borsboom, and Fried 2018).

#### 2.5.3 Analysis of Bayesian Network Model

##### 2.5.3.1 Model Construction

The Bayesian network model was built based on the results of the network analysis. Several alternative model schemes were constructed according to the pre‐established hypothetical model (see Section 1.3), and the best‐fitting model was selected through testing as the final reported result. Model construction and validation is performed by GeNIe 3.0 Academic (64‐bit).

##### 2.5.3.2 Sensitivity Analysis

Sensitivity analysis in GeNIe software was conducted to identify the most influential factors on the target nodes within the Bayesian network model. Trait anxiety and state anxiety were chosen as the target nodes, and the sensitivity analysis results for their parent nodes were calculated to determine the key influencing factors.

##### 2.5.3.3 Model Validity Verification

Data from 303 participants were used for parameter learning, and an additional 305 participants served as the validation dataset to test the model's validity. Predictive accuracy for trait anxiety and state anxiety as target variables was verified through 1000 iterations.

### 2.6 Ethical Considerations

Ethical approval for the study was obtained from the university's institutional review board. Participants were informed of their rights, including their right to withdraw at any point during the study. Data were anonymized, and no personally identifiable information was collected. All data were securely stored following institutional guidelines for confidentiality and privacy protection.

## 3 Result

### 3.1 Descriptive Statistics

**FIGURE 1:**
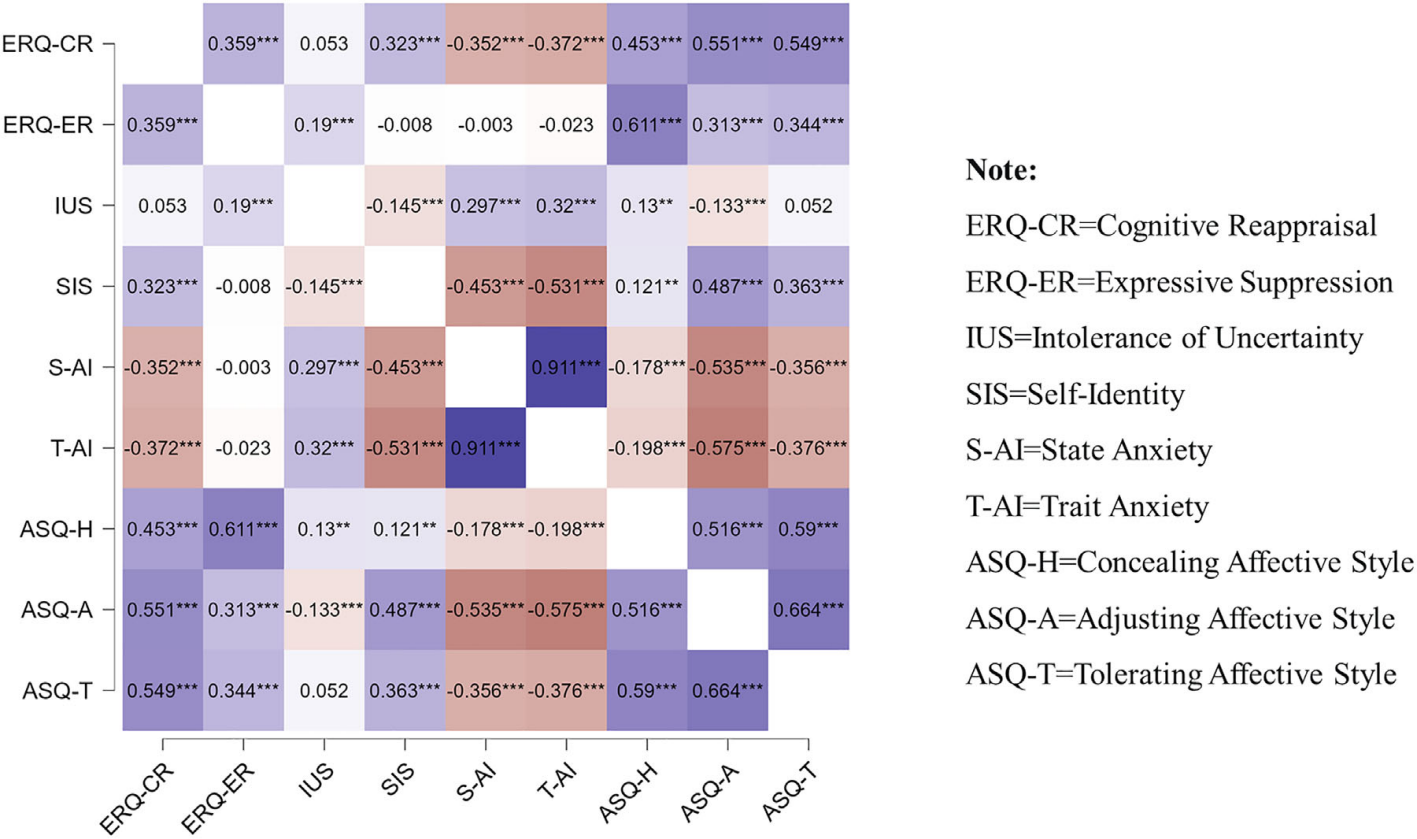
Heatmap of correlations among variables. Brown indicates a negative correlation, with dark brown = *p* < 0.001, medium brown = *p* < 0.01, and light brown = *p* < 0.05. Purple represents a positive correlation, with dark purple = *p* < 0.001, medium purple = *p* < 0.01, and light purple = *p* < 0.05. White indicates a nonsignificant correlation.

Correlation analysis revealed: Trait anxiety showed a significant negative correlation with concealing affective style, adjusting affective style (ASQ‐A), and tolerating affective style within affective style, as well as a significant negative correlation with cognitive reappraisal within emotion regulation; trait anxiety exhibited a significant positive correlation with intolerance of uncertainty within emotion regulation; and trait anxiety displayed a significant negative correlation with personal identity. Additionally, state anxiety and trait anxiety were significantly positively correlated (Figure 1).

### 3.2 Network Analysis

#### 3.2.1 LASSO Network

**FIGURE 2:**
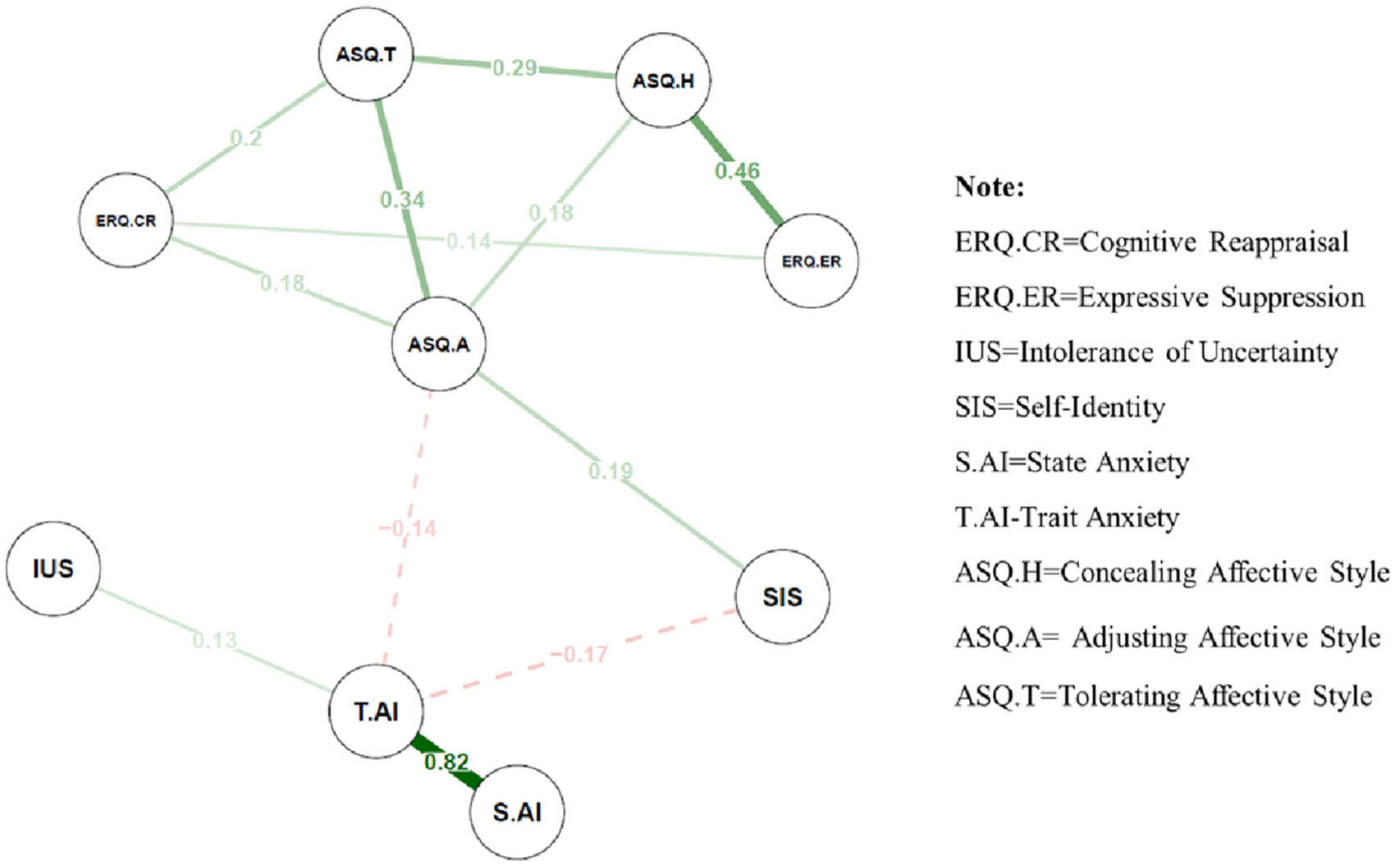
Adaptive LASSO network of factors associated with state anxiety and trait anxiety.

As depicted in Figure 2, the network highlights significant correlations between trait anxiety and several dimensions, including the three dimensions of affective style, two emotion regulation strategies, personal identity, ASQ‐A, and intolerance of uncertainty. Notably, the network also reveals a high degree of correlation between trait anxiety and state anxiety, underscoring a close relationship between these two aspects of anxiety (Figure 2).

#### 3.2.2 Centrality Indices

The mgm package was used to determine the predictability of each node. The predictability of each node refers to, in network analysis, the ability to predict the value of each node (variable) in the network using information from other nodes (see Table 2).

**TABLE 2:** Predictability of each node.

Item	Variable	RMSE	*R* ^2^
1	ERQ.CR	0.768	0.409
2	ERQ.ER	0.764	0.415
3	IUS	0.907	0.176
4	SIS	0.793	0.371
5	S.AI	0.408	0.833
6	T.AI	0.381	0.855
7	ASQ.H	0.662	0.560
8	ASQ.A	0.607	0.631
9	ASQ.T	0.662	0.560

**FIGURE 3:**
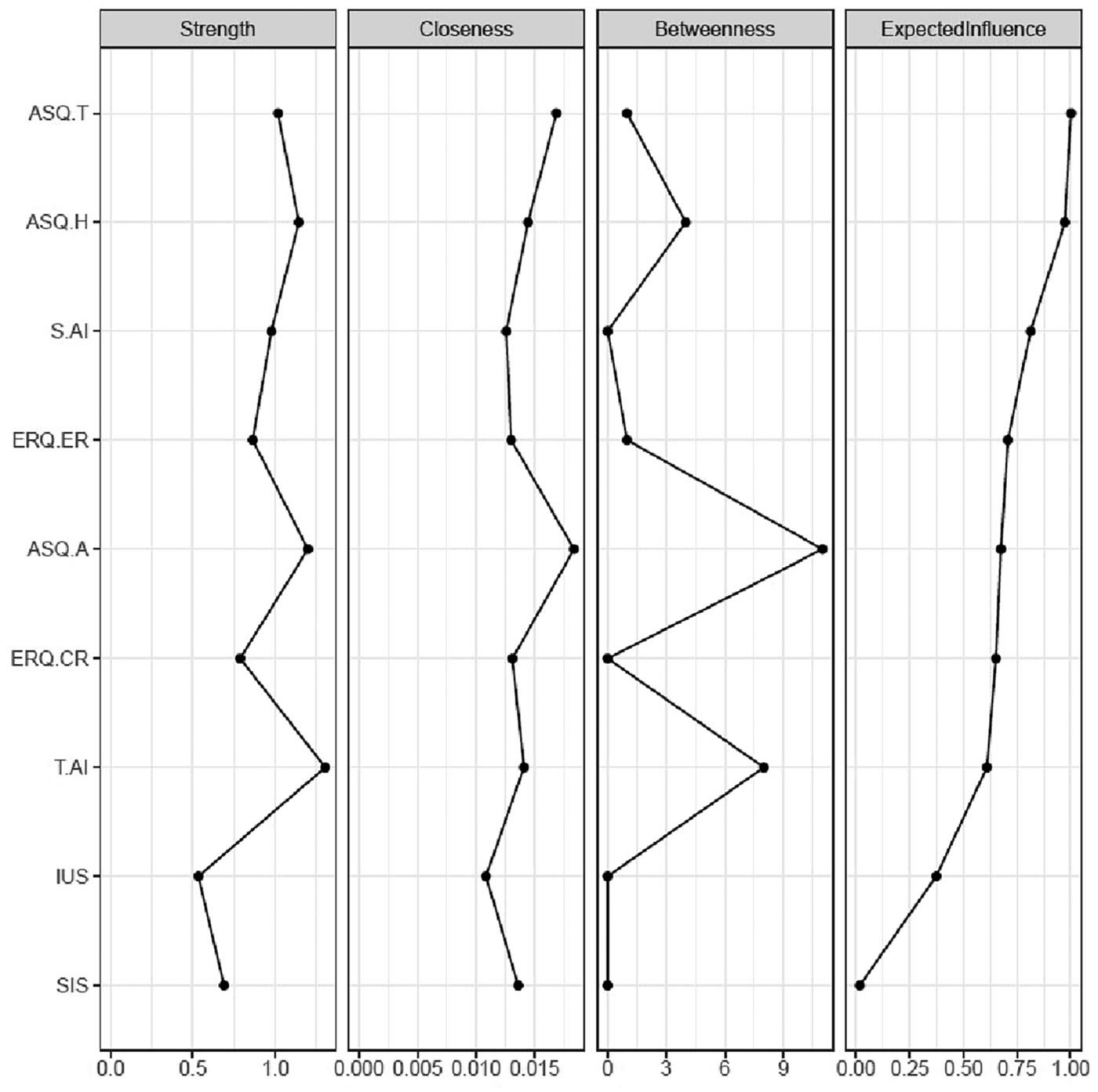
Centrality indices of each factor.

The analysis reveals that the “Adjusting Affective Style” node ranks highly across four key metrics: strength, closeness centrality, betweenness centrality, and expected influence. Both “Tolerating Affective Style” and “Emotion Regulation” also score significantly in three areas: strength, closeness centrality, and betweenness centrality. In contrast, “Self‐Identity” and “Concealing Affective Style” demonstrate predominant influence in terms of strength and closeness centrality (see Figure 3).

Centrality indices were computed to assess the influence of nodes within the network, followed by a thorough stability analysis. The results were promising: betweenness centrality achieved a CS coefficient of 0.283, comfortably exceeding the minimum threshold of 0.25. Closeness centrality demonstrated even greater robustness, with a CS of 0.595, surpassing the critical threshold of 0.5. Additionally, both the strength and expected influence indices exhibited exceptional stability, each with a CS of 0.750, well above the desirable threshold of 0.75.

#### 3.2.3 Robustness

**FIGURE 4:**
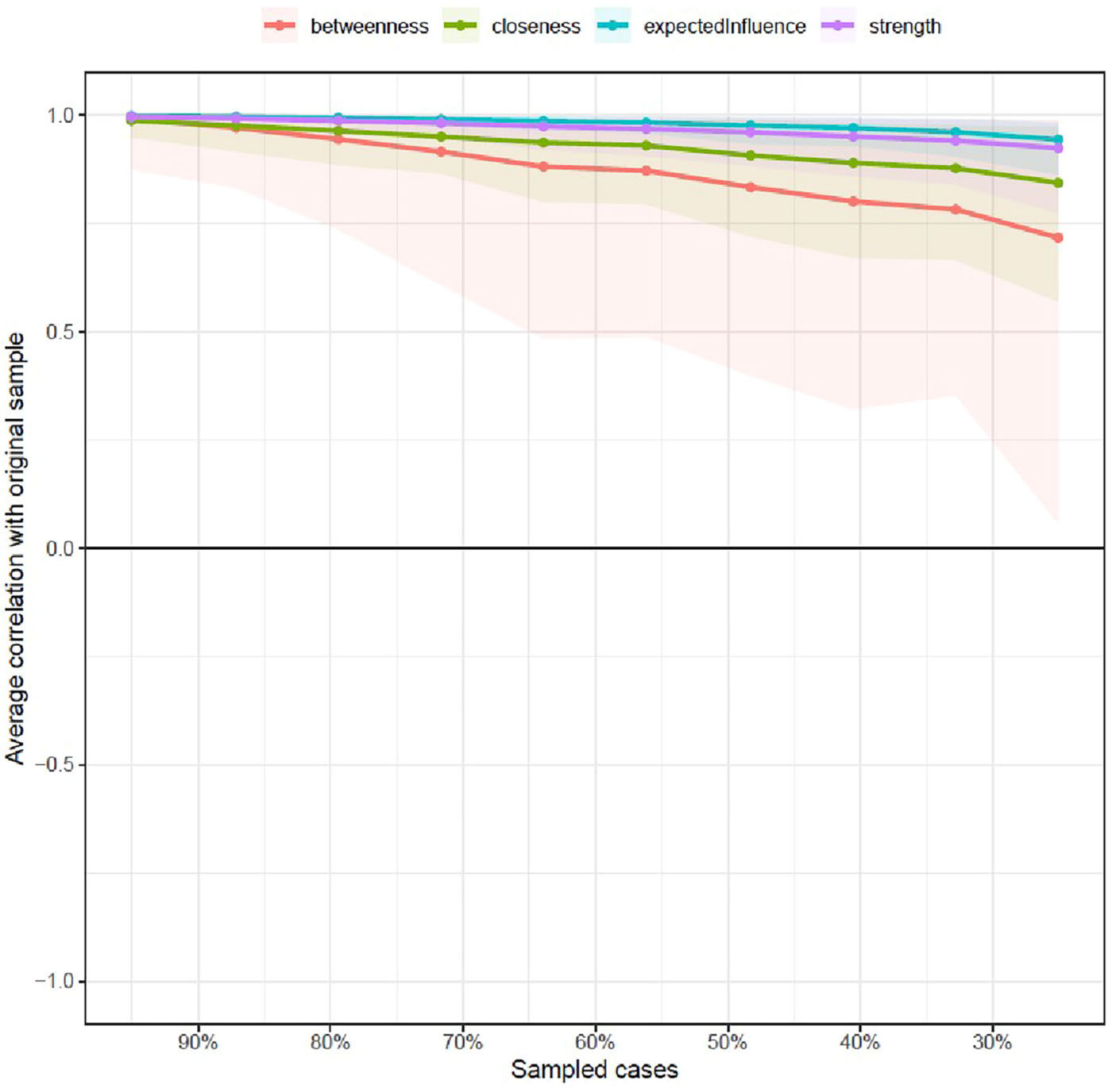
Bootstrapped stability coefficients of centrality indices.

Correlation stability coefficients (CS coefficients) for the centrality indices were calculated to quantify stability and interpretability (Epskamp and Fried 2018). A “case‐dropping subset bootstrap” was conducted using the boot net package in R (Epskamp, Borsboom, and Fried 2018). Centrality correlations greater than 0.7 indicate that centrality was estimated similarly in both the full sample and smaller subsets of the original sample. All indices were estimated with adequate accuracy, ensuring that the network can be meaningfully interpreted (see Figure 4).

### 3.3 Construct Bayesian Network Model

#### 3.3.1 Construction of the Model and Parameters Learning

**FIGURE 5:**
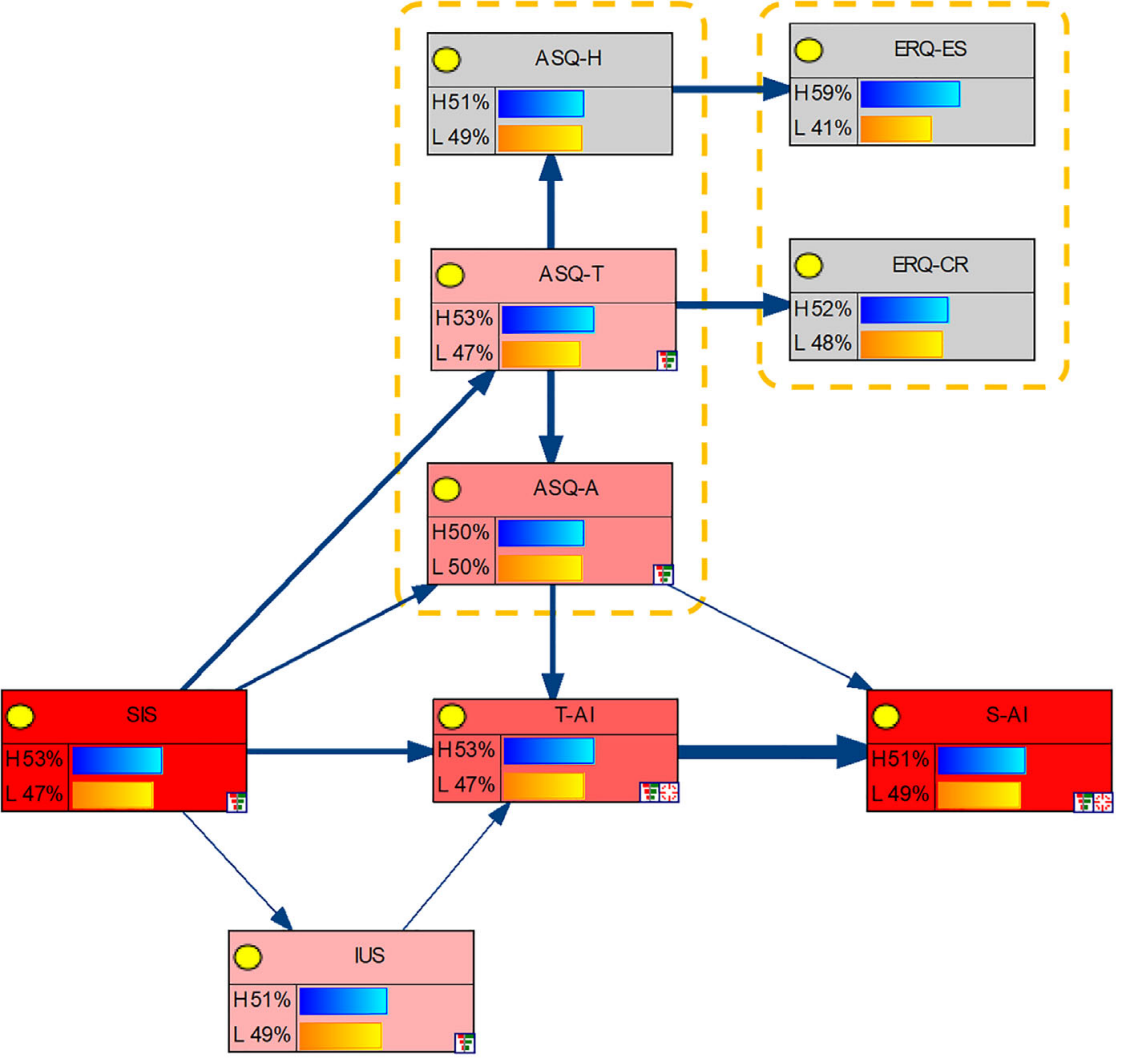
Bayesian network model influencing anxiety levels. ERQ‐CR, cognitive reappraisal; ERQ‐ER, expressive suppression; IUS, intolerance of uncertainty; SIS, self‐identity; SAl, state anxiety; T‐AI, trait anxiety; ASQ‐H, concealing affective style; ASQ‐A, adjusting affective style; ASQ‐T, tolerating affective style.

Based on the initial hypothetical model and the results of the network analysis, the Bayesian network model was constructed as shown in Figure 5. In this model, the two components of anxiety are arranged in a sequential chain, with Trait Anxiety Inventory (T‐AI) serving as a precursor condition influencing State Anxiety Inventory (S‐AI). SIS constitutes a factor affecting T‐AI, with IU acting as a mediator between SIS and T‐AI. The three components of ASQ—ASQ‐H (concealing), ASQ‐T (tolerating), and ASQ‐A (adjusting)—also form a chain‐like relationship. Notably, ASQ‐A directly mediates the relationship from SIS to T‐AI. The two components of the ERQ, ERQ‐ER (expressive suppression) and ERQ‐CR (cognitive reappraisal), are relatively independent. ERQ‐ER is influenced by ASQ‐H, while ERQ‐CR is affected by both ASQ‐T and ASQ‐A. Overall, ERQ does not have a direct relationship with anxiety.

#### 3.3.2 Sensitivity Analysis

The sensitivity analysis results show that when trait anxiety is the target node, the average sensitivity of personal identity is 0.463, intolerance of uncertainty is 0.072, and ASQ‐A is 0.091. When state anxiety is the target node, the average sensitivity of personal identity is 0.35, intolerance of uncertainty is 0.052, ASQ‐A is 0.075, and trait anxiety is 0.091. The corresponding joint sensitivity of multiple factors is shown in Tables 3 and 4.

**TABLE 3:** Sensitivity analysis of parent nodes for trait anxiety.

	SIS high group				SIS low group				
	ASQ‐A high group		ASQ‐A low group		ASQ‐A high group			ASQ‐A low group	
Item	IUS‐12 high group	IUS‐12 low group	IUS‐12 high group	IUS‐12 low group	IUS‐12 high group		IUS‐12 low group	IUS‐12 high group	IUS‐12 low group
T‐AI high group	0.2941	0.1333	0.5862	0.4762		0.5417	0.4118	0.9615	0.8163
T‐AI low group	0.7059	0.8667	0.4138	0.5238		0.4583	0.5882	0.0385	0.1837

**TABLE 4:** Sensitivity analysis of parent nodes for state anxiety.

Item	ASQ‐A high group		ASQ‐A low group	
	T‐AI high group	T‐AI low group	T‐AI high group	T‐AI low group
S‐AI high group	0.8372	0.1101	0.8718	0.1471
S‐AI low group	0.1679	0.8899	0.1282	0.8529

#### 3.3.3 Model Validity Verification

The accuracy of the predictive probabilities is presented in Table 5.

**TABLE 5:** Accuracy of predicting probabilities of trait anxiety and state anxiety.

Item	Trait anxiety	State anxiety
Total	0.901316 (274/304)	0.884868 (269/304)
High group	0.858065 (133/155)	0.894737 (136/152)
Low group	0.946309 (141/149)	0.875 (133/152)

## 4 Discussion

This study explored the relationships between self‐identity, affective style, emotion regulation, intolerance of uncertainty (IU), and anxiety using both network analysis and Bayesian modeling. The findings confirmed that self‐identity is a core predictor of anxiety, while affective style and IU also played important mediating roles. However, there is no direct correlation between emotion regulation and anxiety.

### 4.1 LASSO Network of Factors Related to Anxiety

Figure 2 illustrates the adaptive LASSO network of factors related to anxiety, uncovering several notable patterns. Self‐identity, intolerance of uncertainty (IU), and ASQ‐A are closely linked to trait anxiety, which, in turn, shows a strong connection with state anxiety. Additionally, the dimensions of affective style cluster together with emotion regulation. These findings align with previous research indicating that self‐identity coherence (Oyserman, Smith, and Elmore 2014) and adaptive affective styles (Davidson 1992) are crucial in managing anxiety. The significant connection between trait and state anxiety also supports existing literature highlighting the continuity between these two forms of anxiety (Endler and Kocovski 2001).

The centrality indices further reinforce these findings. Adjusting affective style emerges as a central hub, exhibiting high levels of strength, betweenness, closeness, and expected influence, suggesting its pivotal role in regulating anxiety. This result is consistent with previous research showing that emotional flexibility, a core aspect of adjusting affective style, is associated with better anxiety outcomes (Bonanno and Burton 2013). Similarly, tolerating affective style and concealing affective style demonstrate high strength, betweenness and expected influence, indicating their significant roles within the network. These findings align with prior studies that emphasize the importance of adaptive emotion regulation and affective flexibility in managing anxiety (Aldao, Nolen‐Hoeksema, and Schweizer 2010; Gross 2013b).

While self‐identity shows relatively lower values in some centrality indices, its strong strength and closeness suggest that its influence can quickly spread throughout the network. Moreover, the adaptive LASSO analysis reveals that, apart from state anxiety, self‐identity has the strongest association with trait anxiety. This finding is consistent with constructivist perspectives that highlight self‐identity as a foundational reference for navigating uncertainty and managing anxiety (Kelly 1955).

Interestingly, despite previous research emphasizing the importance of IU in anxiety (Carleton 2016; Grupe and Nitschke 2013), the analysis indicates a weaker link between IU and trait anxiety compared to emotion regulation, affective style, and self‐identity. One possible explanation for this discrepancy could be the nature of the adaptive LASSO network analysis, which is designed to reduce data redundancy and compress variable coefficients. This process may diminish the relative strength of IU by emphasizing the more direct connections between self‐identity, affective style, and anxiety. Additionally, it is possible that in the presence of other impact, the direct impact of IU on anxiety becomes less pronounced, highlighting the need for a more nuanced understanding of its role within the network. Since both IU and self‐identity involve assessments of uncertainty, it is crucial to explore their absolute impact. The subsequent section on causal relationships will further investigate the role of IU in the context of anxiety.

### 4.2 Key Findings From the Bayesian Network Model

The Bayesian network model indicates that the initial hypotheses H1, H2, and H3 are supported, while hypothesis H4 is challenged.

The model shows that the SIS is the primary cause of anxiety, which is consistent with previous research findings (Lindquist, Satpute, and Gendron 2015; Oyserman, Smith, and Elmore 2014) and supports hypothesis H1. Intolerance of uncertainty (IU) also impacts anxiety, a conclusion that aligns with the findings of Carleton (2016) and Grupe and Nitschke (2013). Additionally, IU serves as a mediator between SIS and anxiety, which supports hypothesis H2. The results further indicate that among the three components of affective style, ASQ‐A directly mediates the relationship from SIS to T‐AI, thus supporting hypothesis H3.

However, the results deviate from the initial hypotheses in that emotion regulation is found to be an outcome of affective style, and neither of its subcomponents (cognitive reappraisal and expressive suppression) shows a direct association with anxiety. This finding differs from previous research, which has shown a correlation between emotion regulation and anxiety (e.g., Gross 2013a) and suggested that emotion regulation can alleviate anxiety (e.g., Aldao, Nolen‐Hoeksema, and Schweizer 2010).

Affective style was found to influence emotion regulation. However, emotion regulation did not mediate the relationship between affective style and anxiety, thereby rejecting hypothesis H4.

**FIGURE 6:**
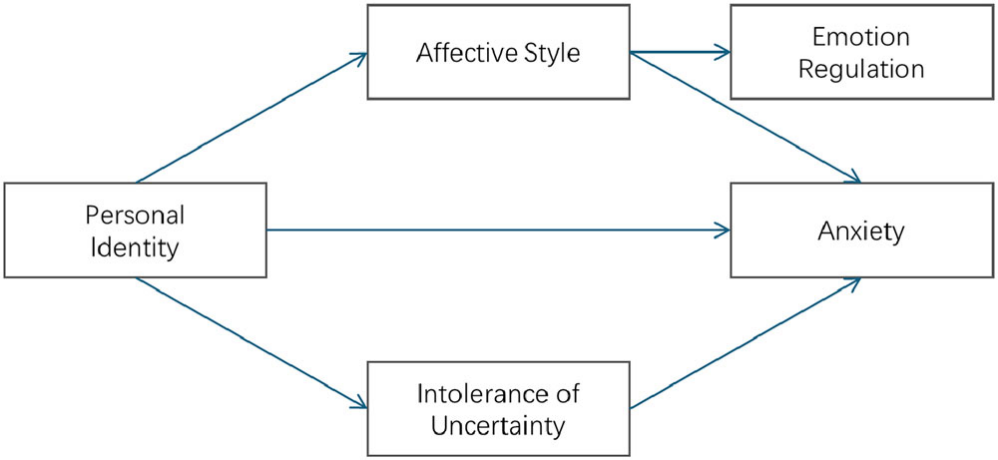
Schema of factors.

In summary, the Bayesian network model analysis led to the mechanism depicted in Figure 6. Here, the SIS serves as the fundamental influencing factor for anxiety, with both affective style and intolerance of uncertainty (IU) acting as mediators between SIS and anxiety. Notably, the ERQ does not serve as a mediator between ASQ and anxiety; instead, it appears as an output of ASQ, operating in parallel with anxiety.

This study indicates the de‐correlation between cognitive reappraisal, expressive suppression, and anxiety as a significant finding. This result suggests that the previously reported relationship between cognitive reappraisal, expressive suppression, and anxiety may actually be a spurious correlation. Both the two strategies and anxiety appear to be influenced by a common underlying factor, which leads to a natural association in their patterns of change. However, there is no direct correlation between the two. This is similar to the positive correlation often observed between ice cream sales and the number of drownings, where both are actually results of rising temperatures (Bollen and Pearl 2013; Pearl 2009).

It is important to note that the Bayesian network model constructed in this study achieved a fit of 0.88, which may raise concerns about potential overfitting. In response, we emphasize that this study is strictly theory driven. Before data collection, the researchers developed a robust hypothetical model based on an extensive literature review and theoretical analysis, grounded in well‐established theoretical and empirical evidence. As a result, the subsequent data collection and computational outcomes that support the initial hypothesis reflect a validation process aligned with scientific research standards, rather than an overfitting result produced by post hoc data manipulation techniques.

### 4.3 Causal Inference Based on Bayes

While cross‐sectional data presents challenges for causal inference, we employed adaptive LASSO network analysis and Bayesian network analysis to explore complex relationships within multivariate environments. These methods were selected for their robust capabilities in interpreting complex relationships that are often difficult to capture with traditional statistical approaches. Notably, this approach aligns with other causal inference, as both rely on the principles of probability theory.

Bayesian network analysis is particularly valuable as it integrates prior knowledge with current data to infer posterior probabilities of potential causal models. Although not a tool for establishing causality definitively, it provides a framework for formalizing and testing causal hypotheses through probability theory. This approach allows us to assess the relative likelihood of various hypothetical models, even in the absence of time series data, especially when supported by existing theory or prior knowledge.

Our research emphasizes the probabilistic nature of model predictions rather than definitive conclusions. While this method has been widely used in engineering for risk prediction and management, its application in psychology remains limited. Through this study, we aim to expand the methodological toolkit in psychology, particularly for analyzing complex causal relationships and predictive modeling.

### 4.4 Clinical Significance

The findings of this study have several important implications for clinical practice.

First, given the central role of self‐identity in anxiety, interventions that strengthen an individual's sense of self, such as Acceptance and Commitment Therapy (Hayes, Strosahl, and Wilson 2011), may be particularly effective in reducing anxiety symptoms.

Second, while intolerance of uncertainty (IU) played a mediating role, suggesting that uncertainty‐focused interventions like cognitive flexibility training (Hirsch et al. 2018) could be beneficial, especially for individuals with generalized anxiety disorder (Carleton 2016).

Finally, our findings support the integration of emotional flexibility approaches, such as emotion‐focused therapy (Greenberg 2015), while indicating that relaxation‐focused interventions alone may be insufficient for anxiety treatment (Conrad and Roth 2007).

### 4.5 Limitations and Future Directions

Despite its contributions, this study has several limitations. First, the reliance on self‐report data introduces potential biases, such as social desirability or participants’ limited self‐awareness. Future research should incorporate objective measures, such as physiological or behavioral assessments, to validate the findings.

Second, this study examined the general mechanisms of anxiety in ordinary participants. Future research could focus on specific participants, such as individuals with anxiety disorders, to further test the validity of the psychological mechanism model developed in this study for explaining anxiety symptoms in special populations.

Lastly, the study did not account for external factors such as social support or environmental stressors, which are known to influence anxiety. Future research should incorporate these variables into the model to provide a more comprehensive understanding of anxiety.

## 5 Conclusion

Based on this research, we have drawn the following conclusions: (1) self‐identity, affective style, and intolerance of uncertainty directly predict trait anxiety; (2) affective style and intolerance of uncertainty act as mediators between SIS and anxiety; (3) emotion regulation strategies are the outcome of affective style and run parallel to anxiety, with no direct correlation between the two.

## Author Contributions

**Ruizhi Huang**: methodology, writing–original draft, writing–review and editing, software. **Huiqing Shen**: investigation, visualization, software, formal analysis. **Ye Yuan**: methodology, investigation. **Ke Jiang**: project administration, conceptualization, resources, writing–review and editing, supervision. **Zhilin Wang**: investigation, project administration, data curation.

### Peer Review

The peer review history for this article is available at https://publons.com/publon/10.1002/brb3.70290.

## Data Availability

The data that support the findings of this study are available from the corresponding author upon reasonable request.
